# Synbiotic combination of fructooligosaccharides and probiotics ameliorates the metabolic dysfunction-associated steatotic liver disease

**DOI:** 10.71150/jm.2411002

**Published:** 2025-02-27

**Authors:** Sang Yoon Lee, Su-Been Lee, Goo-Hyun Kwon, Seol Hee Song, Jeong Ha Park, Min Ju Kim, Jung A Eom, Kyeong Jin Lee, Sang Jun Yoon, Hyunjoon Park, Sung-Min Won, Jin-Ju Jeong, Ki-Kwang Oh, Young Lim Ham, Gwang Ho Baik, Dong Joon Kim, Satya Priya Sharma, Ki Tae Suk

**Affiliations:** 1 Institute for Liver and Digestive Diseases, Hallym University, Chuncheon 24253, Republic of Korea; 2 Department of Nursing Daewon University College, Jecheon 27135, Republic of Korea; 3 Department of Internal Medicine, Hallym University College of Medicine, Chuncheon 24253, Republic of Korea

**Keywords:** synbiotics, prebiotics, probiotics, Metabolic dysfunction-associated steatotic liver disease (MASLD), gut microbiome

## Abstract

Synbiotics have become a new-age treatment tool for limiting the progression of metabolic dysfunction-associated steatotic liver disease; however, inclusive comparisons of various synbiotic treatments are still lacking. Here, we have explored and evaluated multiple synbiotic combinations incorporating three distinctive prebiotics, lactitol, lactulose and fructooligosaccharides. Of the synbiotic treatments evaluated, a combination of fructooligosaccharides and probiotics (FOS+Pro) exhibited superior protection against western diet-induced liver degeneration. This synbiotic (FOS+Pro) combination resulted in the lowest body weight gains, liver weights and liver/body weight ratios. The FOS+Pro synbiotic combination substantially alleviated liver histopathological markers and reduced serum AST and cholesterol levels. FOS+Pro ameliorated hepatic inflammation by lowering expression of proinflammatory markers including TNF-α, IL-1β, IL-6, and CCL2. FOS+Pro significantly improved steatosis by restricting the expression of lipid metabolic regulators (ACC1, FAS) and lipid transporters (CD36) in the liver. These findings are critical in suggesting that synbiotic treatments are capable of restraining western diet-induced metabolic dysfunction in the liver. Additionally, this study demonstrated that adding probiotic strains amplified the effectiveness of fructooligosaccharides but not all prebiotics.

## Introduction

Previous clinical and preclinical empirical studies showed that dysbiotic gut ecology altered metabolic (Hsu and Schnabl, 2023; Sharma et al., 2024; Tilg et al., 2022) and immunological (Wang et al., 2021) profiles and aggravated various liver disorders, including metabolic dysfunction-associated steatotic liver disease (MASLD) (Bauer et al., 2022; Jennison and Byrne, 2021). Dysbiotic gut ecosystems increased intestinal permeability, which amplified translocation of bacteria and bacterial metabolites to the liver and was a major contributor to the liver inflammation that augmented MASLD progression. Restoration of gut microbial homeostasis was identified as an unconventional therapeutic strategy that showed tremendous potential for controlling MASLD (Sharpton et al., 2021; Sorbara and Pamer, 2022). This unorthodox therapeutic strategy primarily includes the use of prebiotics (nondigestible food which promotes growth of beneficial microbes), probiotics (beneficial microbes), postbiotics (health-promoting microbial byproducts), and synbiotics (combinations of pre- and probiotics) (Lee et al., 2021; Pan et al., 2024; Rong et al., 2023). These unconventional interventions showed promising results for the management of MASLD through amelioration of diverse pathophysiological pathways (Lang and Schnabl, 2020).

In synbiotic treatment strategies, probiotics and prebiotics work synergistically to modulate the gut microbial ecology and alleviate liver degeneration. The prebiotics used most widely in the management of liver diseases are lactitol, lactulose, and fructooligosaccharides (FOS), with considerably promising outcomes. Lactitol is a lactose-based sugar alcohol used to improve liver function and reduce endotoxin levels in the blood of cirrhosis patients (Chen et al., 2013; Lu et al., 2021). Lactulose is a synthetic disaccharide that improves gut microbial diversity by increasing the abundance of short-chain fatty acid (SCFA)-producing bacteria (Bloom and Tapper, 2023; Cutler et al., 2022; Odenwald et al., 2023). FOS is a plant-based oligosaccharide that improves gut dysbiosis and short-chain fatty acid concentrations, improving hepatic health (Huang et al., 2023; Takai et al., 2020). Interestingly, synbiotic treatment strategies, such as combinations of FOS and *B. longum*, used in multiple clinical trials have been shown to reduce liver fat and AST (Malaguarnera et al., 2012). Similarly, synbiotic treatment with FOS and a combination of multiple probiotic strains showed decreases in liver stiffness and inflammatory markers in MASLD (Jin et al., 2021; Loman et al., 2018; Naghipour et al., 2023). These clinical findings underline the targeted use of synbiotics to modulate the intestinal microbiome as a promising therapeutic approach for MASLD management.

Despite the promising potential of synbiotics for modulating gut microbiota and improving MASLD and other liver conditions, several limitations persist in selecting the optimal combination of probiotic strains with lactulose, lactitol, or FOS. Inadequate information about optimal combinations of prebiotics and probiotics is one constraint among several limiting factors. Considering the scarcity of literature defining appropriate synbiotic combinations for well-known prebiotics and probiotics, we have designed a MASLD animal model study using three prebiotics (lactulose, lactitol, or FOS) combined with probiotic strains. We have comprehensively evaluated which synbiotic combinations effectively limited MASLD progression. In this study we have also explored whether adding the probiotics amplified prebiotic effects and which prebiotic effects were augmented by the probiotics.

## Materials and Methods

### Human trial

The participants included in this study either were admitted to the Department of Liver Diseases at Hallym University Hospital or visited for regular health checkups between April 2022 and March 2023 (ClinicalTrials.gov NCT04339725, IRB No. 2016-134). A total of 50 participants were enrolled in this study and were grouped into healthy control patients (HC, n=18), MASLD patients (n=18), and MASH patients (n=14). All the included participants were properly informed and signed a consent form before inclusion. Institutional ethical committee approval was obtained for this trial (IRB No. 2016-134), and 1975 Helsinki Declaration guidelines were strictly followed.

MASLD was characterized following the latest European Association for the Study of the Liver clinical practice guidelines, which include hepatic steatosis combined with one or more cardiometabolic risk factors (European Association for the Study of the Liver (EASL); European Association for the Study of Diabetes (EASD); European Association for the Study of Obesity (EASO), 2024; Kim et al., 2023). Hepatic steatosis was defined by imaging (ultrasound or computed tomography scan) or liver biopsy along with increased liver enzyme levels, such as aminotransferase (AST) or aspartate aminotransferase (ALT) ≥ 50 IU/L. Blood and fecal samples were collected from the participants at the time of enrollment in the study. Patients with high alcohol consumption (males >30 and females >20 g/day), autoimmune disorders, pancreatitis, hemochromatosis, viral liver disease, pregnancy, Wilson’s disease, drug-induced liver injury, or cancers were excluded from this study. Participants who visited the hospital for routine health checkups were included in the HC group. None of the included participants received any treatment by which gut microbial ecology could have been altered. The baseline patient characteristics are presented in Table 1.

### Fecal microbiome analysis

After enrollment of all the participants, fecal samples were collected and stored at -80°C until fecal Genomic DNA. The V3-V4 variable region based 16S rRNA amplicon sequencing was performed from collected participant’s fecal samples as described previously (Sharma et al., 2024). Total of 200mg fecal samples from each participant were used for genomic DNA extraction by using QIAamp stool kit (Qiagen, Germany) and sequence libraries were prepared with NEBNextUltra II FS DNA Library Prep Kit used for Illumina (NewEngland BioLabs, USA). The libraries were quantified by Qubit dsDNA HS assay kit (ThermoFisher Scientific, USA), confirmed by qPCR with KAPA SYBR FAST qPCR Master Mixkit (Kapa Biosystems, USA) and quality assessed on Bioanalyzer 2100 (Agilent, USA) using a DNA 12,000 chip. The liberies sequencing was done by using NovaSeq 6000 platform (Illumina, USA) paired with end (PE) 150 bp reader.

In sequencing, extracted fecal DNA were amplified for V3-V4 variable region of 16S rRNA bacterial gene and attached with barcoded fusion primers. The attached forward primer had p5 adapter, i5 index, and gene specific primer 341F (5′-AATGATACGGCGACCACCGAGATCTACAC-XX XXXXXX-TCGTCGGCAGCGTCAGATGTGTATAAGAGACAG-CCTACGGGNGGCWGCAG3′; X indicates the barcode region), and the reverse fusion primer attached with the p7 adapter, i7 index, and gene-specific primer 805R(5′-CAAGCAGAAGACGGCATACGAGATXXXXXXXXGTCTCG TGGGCTCGGAGATGTGTATAAGAGACAG-GACTACHVGGGTATCTAATCC-3′), combined with sequencing adapters and dual-index barcodes for the Nextera XT kit (Illumina, USA). The C1000 touch thermo-cycler (Bio-Rad Laboratories, Inc., USA) used for PCR amplification with following conditions: denaturation at 95°C for 3 min; followed by 25 cycles of denaturation at 95°C for 30 s, annealing at 55°C for 30 s, extension at 72°C for 30 s and final extension at 72°C for 5 min. The 1% agarose gel electrophoresis and Gel Doc XR+ imaging system (Bio-Rad Laboratories, Inc., USA) used for the confirmation of the amplified PCR product. Then PCR product was purified by Agencourt AMPure XP beads (Beckman Coulter, USA) and library was constructed and assessed on a Bioanalyzer 2100 (Agilent, USA) using a DNA 12000 chip and quantified by qPCR with a KAPA SYBR FAST qPCR Master Mix kit (Kapa Biosystems, USA). The EzBioCloud Apps (CJ Bioscience Inc., Korea) was used for Microbial Taxonomic profiling and comparative analysis. Assignment of OTUs at species level for each read were performed using CJ bioscience’s 16S rRNA database (DB ver. PKSSU4.0) (Yoon, 2017 #358) and sequence similarity determined in UNCLUST and CHDIT (Edgar, 2010 #359). The alpha-diversities (Chao1, ACE, Jackknife, Simpson, Shannon, NPShannon) and Beta-diversities (PCoA and UPGMA) were by using MTP analyzer and all data is available on the ChunLab’s EzBioCloud.

### Animal experiments

Six-week-old male C57BL/6J specific pathogen-free mice were purchased from Doo-Yeol Biotech (Seoul, Korea) and housed in conditions of 22 ± 2°C in 40–50% humidity with a 12-hour light/dark cycle. After one week of acclimatization, the mice were randomly divided into 8 experimental groups (n=9): normal diet control (NC), western diet control (WC), lactitol (LAC), lactitol + probiotic strains (LAC+ Pro), lactulose (LAT), lactulose + Pro (LAT+ Pro), fructooligosaccharides (FOS), and fructooligosaccharides + Pro (FOS + Pro). NC group mice were fed a normal chow diet (2018S, Teklad Global), and all other mice were fed a western diet with adjusted calories (TD.88137, Teklad Global). All the mice were fed their respective diets for 15 weeks and had unrestricted access to food and water throughout the experiment. The animal experimental plan was reviewed, revised and approved by the animal experimental management committee of Hallym University College of Medicine (2022-25), and the guidelines issued by the National Institute of Health for the Care and Use of the Animals were strictly followed.

### Prebiotics and probiotic treatment

Prebiotics for this animal experiment includes lactitol (Yuhan Corporation, South Korea), lactulose (JW Pharmaceutical, Korea), and fructooligosaccharides (F8052-50g, Sigma Aldrich, USA), which were orally given with or without probiotic strains to their respective groups for three time in a week with dosage concentration of 5g/kg/ dosage, until 15 weeks of duration. We have used 6 bacterial strains including 4 lactobacillus species (*L. acidophilus* CKDB007, *L. reuteri* CKDB019, *L. fermentum* CKDB004, *L. lactis* CKDB001), and 2 bifidobacterium species (*B. breve* CKDB002, *B. longum* CKDB004). These probiotic strains were isolated from various sources as followed; *L. reuteri* CKDB019: isolated from maternal colostrum, *L. fermentum* CKDB004: isolated from infant saliva, *L. lactis* CKDB001: isolated from fermented milk, *L. acidophilus* CKDB007, *B. breve* CKDB002, and *B. longum* CKDB004 isolated from infant feces. Then after isolation, strains were grown anaerobically in de Man, Rogosa and Sharpe medium (BD/Difco) at 37°C for 24 h. All the bacterial strain were stocked at -80°C in 20% skim milk solution with concentration of 1×10^9^ CFU/ml. Before oral gavage, a cocktail solution having equal number of CFU for probiotic strains (each bacterial strain added with approximately 1.67x10^8^ CFU/ml concentration) was prepared and total of 1x10^9^ CFU/mouse was orally administered to the respective groups.

### Serum biochemical analysis

At the end of 15^th^ week all groups’ mice were weighted and exposed to isoflurane and blood samples were collected from hepatic portal vain in an untreated Eppendorf tube. Serum samples were separated from blood by centrifugation for 15 min at 1,500 g at 4°C and collected serum used for alanine aminotransferase (ALT), aspartate aminotransferase (AST) and total cholesterol analysis.

### Liver histological analysis

After sacrifice, mice liver was isolated and weight, appearance and surface color data were collected. Then liver sections were cut, fixed in 10% formalin. Then fixed liver tissues were embedded in paraffin, and the liver sections were prepared for histological staining (hematoxylin and eosin). All liver histology samples were assessed by a group’s blinded pathologist. Liver histology samples were evaluated for Metabolic dysfunction associated steatohepatitis (MASH) based on steatosis, lobular inflammation, and ballooning scoring using NAS-based guidelines. The steatosis graded from 0 to 3 according following the Clinical Research Network scoring system (0: <5%, 1: 5%–33%, 2:34%–66%, and 3: >66%). Similarly, inflammation also graded from 0 to 3 (0: none, 1: 1–2 foci per × 20field, 2: 2–4 foci per × 20 field, 3: >4 foci per × 20 field).

### Liver RNA extraction and qReal-Time PCR

The TRIzol Reagent (Thermo Fisher Scientific) used for total liver RNA extraction by following the kit manual. Total RNA was quantified using spectrophotometer and 1 μg RNA from each sample was used for cDNA preparation by High-Capacity cDNA Reverse Transcription Kit (Thermo Fisher Scientific) using SimpliAmp™ Thermal Cycler PCR System (Thermo Fisher Scientific).

Quantitative real-time PCR (qPCR) was done using PowerUp™ SYBR™ Green Master Mix (Applied Biosystems, USA) and Step-One Plus equipment (Applied Biosystems, USA). The qPCR was performed using the following conditions, initial step at 95°C for 1 min, followed by 40 cycles of 95°C for 15 sec, 60°C for 15 sec, with a final extension at 60°C for 1 min. To measure the relative mRNA expression levels *Cyclophilin A (PPIA)* gene was used as internal normalization control. The gene primer sequence used in this study for the relative analysis is listed in Table 2.

### Statistical analysis

#### Human data

Group-based statistical analysis and graphical presentation of human fecal metagenomics data were performed using GraphPad Prism (version 9.5.1). Group comparisons between the mean values of more than 2 groups were evaluated with nonparametric one-way ANOVA or the Kruskal‒Wallis test with Dunn’s post hoc test. Comparisons between two groups were evaluated using nonparametric Student’s t test or the Mann–Whitney–Wilcoxon rank-sum test.

#### Animal data

Data acquired from animal experiments were analyzed and graphically represented with GraphPad Prism (version 9.5.1). Initially, comparisons of the means between the different animal experimental groups for each parameter were performed using ordinary one-way analysis of variance (ANOVA) with Tukey's post hoc test. Furthermore, an unpaired parametric student’s t test was also employed in second step to evaluate and compare the means difference of two particular groups.

## Results

### Significant declines in *Lactobacillus* and *Bifidobacterium* genera abundances were associated with dysbiosis in MASLD

**Fig. 1.**
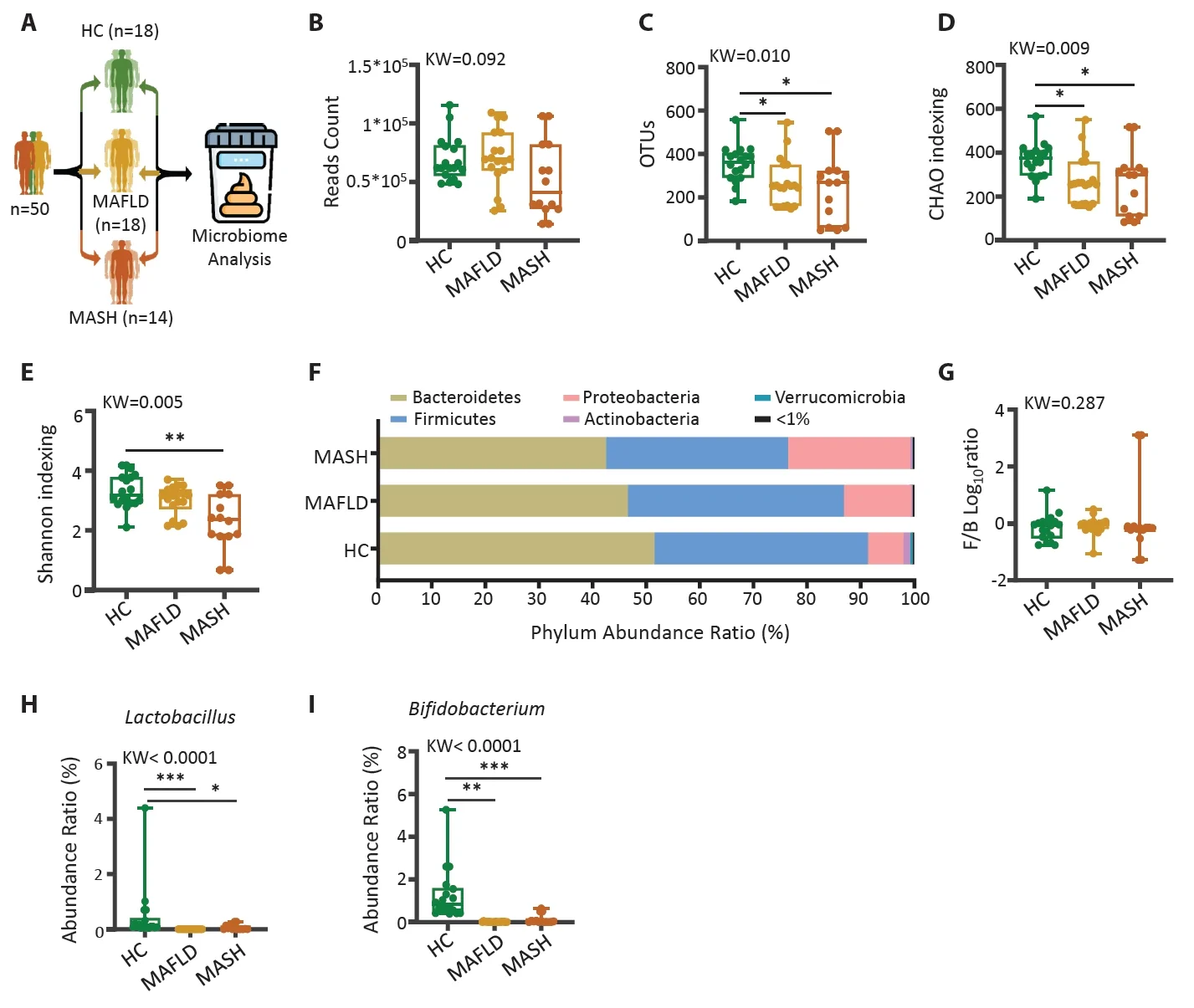
Human gut microbiome profiling showed *Lactobacillus* and *Bifidobacterium* genus dependent dysbiosis in Metabolic Associated Liver Diseases (MASLD). (A) Human trial flow, (B) Target Reads count in MASLD groups, (C) OTUs in MASLD groups, (D) CHAO indexing in MASLD groups, (E) Shannon indexing in MASLD groups, (F) Phylum abundance ratio in MASLD groups, (G) F/B log_10_ ratio in MASLD groups, (H) *Lactobacillus* genus abundance in MASLD groups, (I) *Bifidobacterium* genus abundance in MASLD groups. Statistical change in mean among the MASLD groups calculated by ANOVA using Kruskal-Wallis sum-rank test (KW) and represented by; *p<0.05, **p=0.001, ***p>0.0001.

Comparative analysis of human gut microbiomes showed dysbiosis of the gut ecosystems in MAFLD and MASH patients (Fig. 1). Fecal samples collected from HC, MAFLD and MASH patients (Fig. 1A) showed no significant differences in the total selected read counts (Fig. 1B). However, the number of operational taxonomical units (OTUs) significantly declined as MASLD severity increased (Fig. 1C). The severity-associated declines in OTUs and unchanged read counts directly influenced diversity indexing. The alpha diversity indices, such as CHAO and Shannon indices, showed severity-dependent, significant declines in MASLD patients (Fig. 1D, E). Phylum-level abundances of Bacteroidetes declined and Proteobacteria showed increased in association with MASLD progression (Fig. 1F). Firmicutes showed varied and unchanged abundance ratios in HC and MAFLD but declined in MASH patients, which presented a rising F/B ratio trend (Fig. 1G). Abundances of *Lactobacillus* and *Bifidobacterium* genera showed declines associated with progression of disease severity in MASLD patients (Fig. 1H, I).

### Synbiotic treatment with a combination of fructooligosaccharides and probiotics restricted MASLD-associated phenotypical changes in mice

**Fig. 2.**
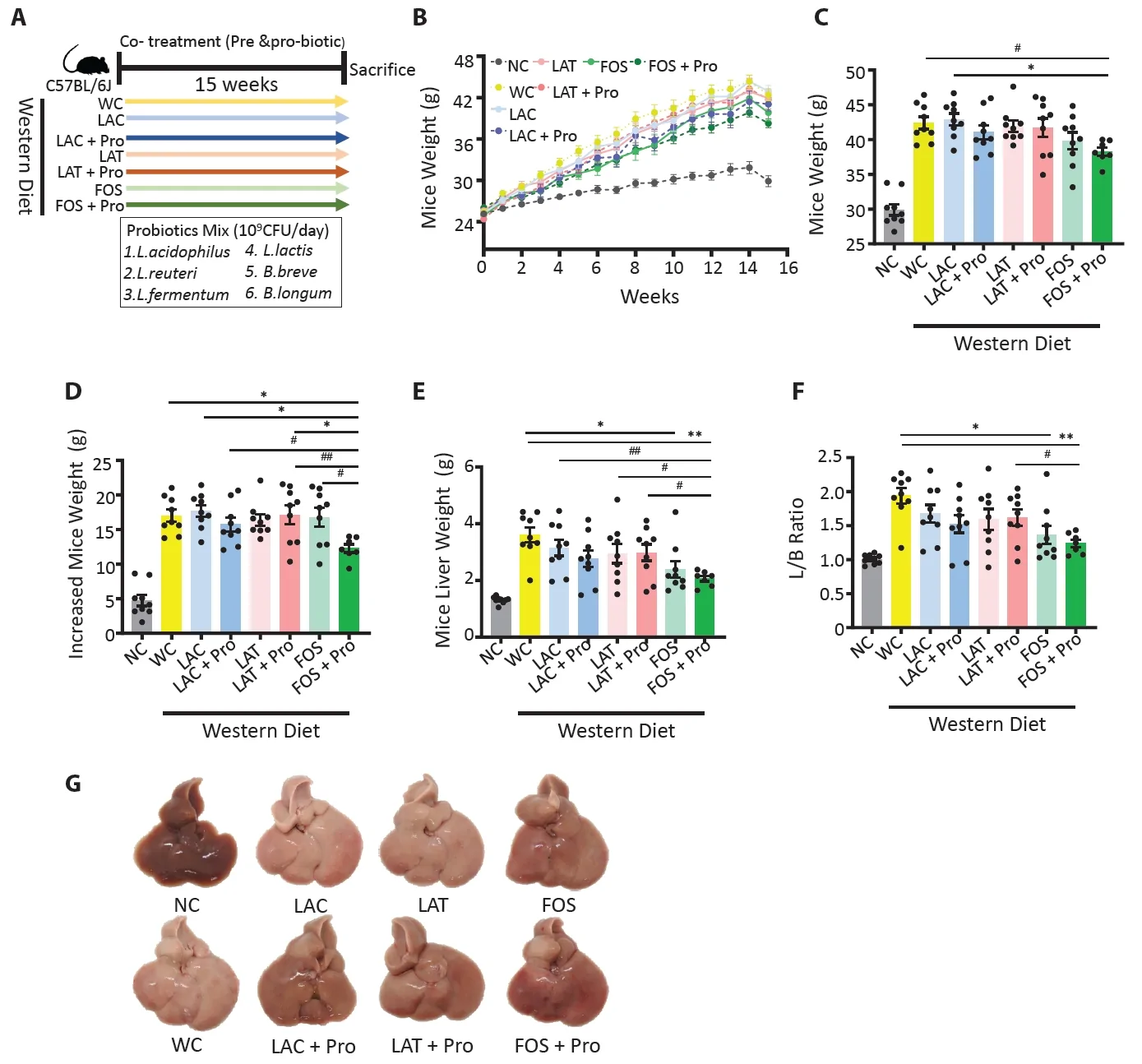
Synbiotic treatment with combination of fructooligosaccharides and probiotic restricted MASLD associated phenotypical changes in mice. (A) Diagrammatic presentation of western diet animal experiment, (B) Weekly animal body weight in all experimental groups, (C) Animal body weight at the end of the experiment, (D) Increment in animal bodyweight in all the experiment groups, (E) Animal liver weight, (F) liver/body weight ratio, (G) Representative image of the liver in all experimental groups. Animal data showed as mean ± SEM and difference between the mean was analyzed by ANOVA with tukey's post hoc test and represented by; *p<0.05, **p<0.001, ***p<0.0001. The mean difference between the two specific groups were analyzed with unpaired parametric student’s t-test and represented by #*p*<0.05, ##*p*<0.01, ###*p*<0.001.

To evaluate synergetic effects of prebiotics and probiotics in mice with diet-induced metabolic-associated liver diseases, we fed mice a western-diet feed for 15 weeks (Fig. 2A). For this experiment, we used 3 prebiotics, lactitol (LAC), lactulose (LAT), and fructooligosaccharides (FOS), which have each shown promising beneficial effects in patients with liver diseases. Based on the above in-house human gut microbiome data, which showed significant severity-associated declines in abundances of *lactobacillus* and *bifidobacterium* genera in the MASLD patients, we selected 4 bacterial strains from the *lactobacillus* genus (*L. acidophilus*, *L. reuteri*, *L. fermentum*, and *L. lactis*) and 2 bacterial strains from the *bifidobacterium* genus (*B. breve* and *B. longum*) for this animal experiment. These bacterial strains were fed with the lactitol + probiotics (LAC+ Pro), lactulose + probiotics (LAT+ Pro), and fructooligosaccharides + probiotics (FOS + Pro) to mouse groups at a concentration of 10^9^ CFU/ml/mouse three times a week. The treatment groups presented a variety of weight differences when compared to the western diet control group (Fig. 2B-D). The LAC mice showed similar weights to the western diet control group (WC), while LAC+ Pro, LAT, LAT+ Pro, and FOS mice showed slightly lower body weights compared to the WC group. Interestingly, the FOS + Pro group showed significantly lower bodyweight compared to WC and LAC mice at the end of the experiment. The increased-weight data showed that FOS + Pro-group mice gained the smallest amount of weight, with significantly lower bodyweights than all the other western-diet-fed mice. The FOS + Pro mice showed 38.6%, 44.1%, 28.2%, 33.6%, 39.7%, and 36.8% less weight gains than WC, LAC, LAC+ Pro, LAT, LAT+ Pro, and FOS mice, respectively. The FOS + Pro mice showed significantly lower liver weights and other phenotypical parameters compared to the other western-diet-fed mice (Fig. 2E-G). The FOS + Pro mice showed 74.8%, 52.9%, 33.8%, 42.7%, 44.6%, and 16% lower liver weights compared to WC, LAC, LAC+ Pro, LAT, LAT+ Pro, and FOS mice, respectively (Fig. 2E). Additionally, the FOS + Pro mice showed significantly lower liver/body weight ratios (L/B ratio) compared to the other western-diet-fed mice (Fig. 2F). The FOS + Pro mice showed 56.6 %, 35.2%, 23.2%, 28.6%, 30.6%, and 10.4% lower L/B ratio compared to WC, LAC, LAC+ Pro, LAT, LAT+ Pro, and FOS mice, respectively.

### Synbiotic treatment with a combination of fructooligosaccharides and probiotics improved MASLD-associated clinical markers in mice

**Fig. 3.**
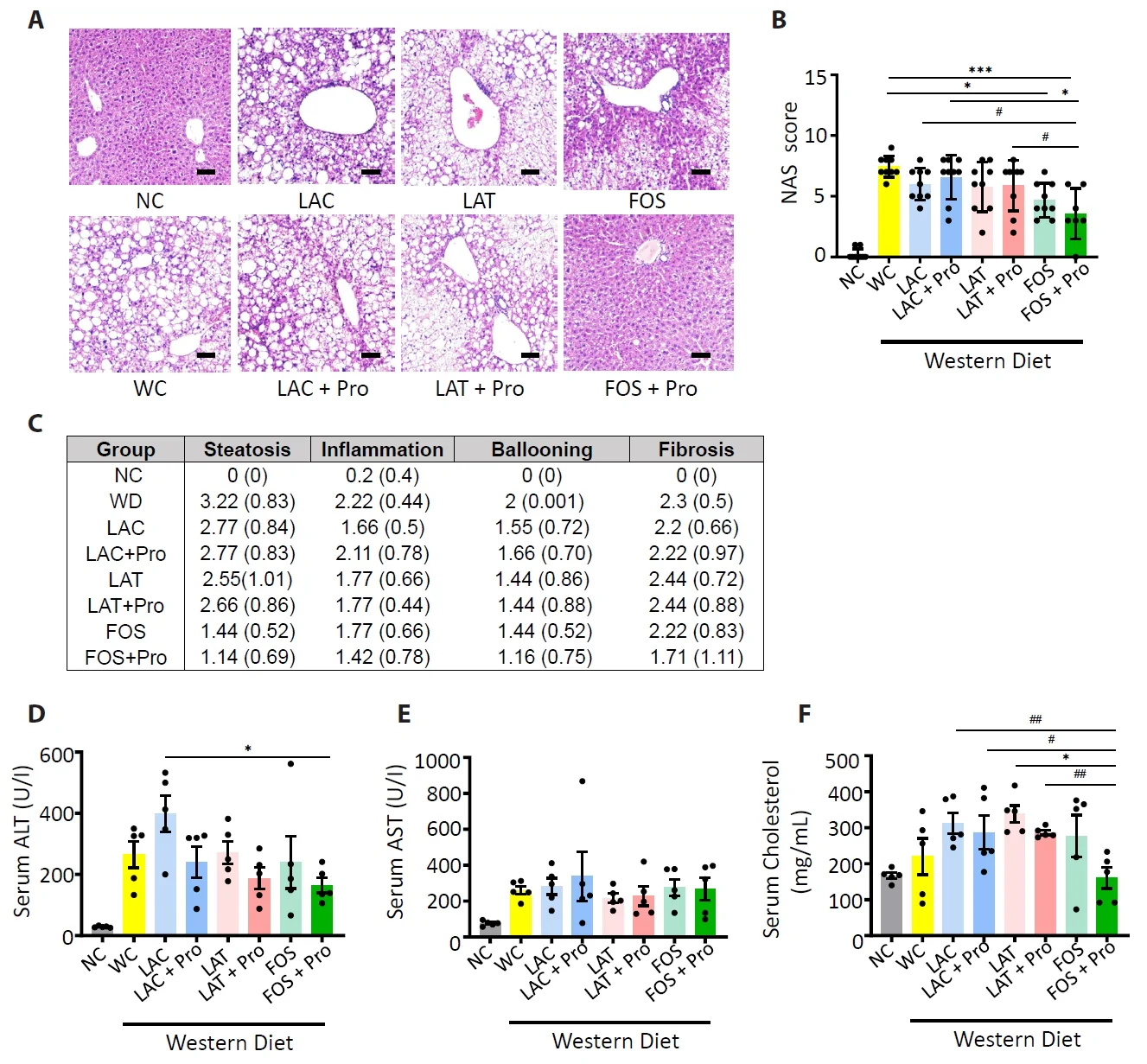
Synbiotic treatment with combination of fructooligosaccharides and probiotic improved MASLD associated markers in mice. (A) Liver H&E stain, (B) MASH score evaluated by H&E stain, (C) Histopathological analysis, (D) Serum ALT level, (E) Serum AST level, (F) Serum Cholesterol level. Animal data showed as mean ± SEM and difference between the mean was analyzed by ANOVA with tukey's post hoc test and represented by; *p<0.05, **p<0.001, ***p<0.0001. The mean difference between the two specific groups were analyzed with unpaired parametric student’s t-test and represented by #*p*<0.05, ##*p*<0.01, ###*p*<0.001.

Western dietary intake creates MASLD-associated pathophysiological changes in the liver, which are evaluated by a combination of histology and clinical blood markers (Fig. 3A-F). Therefore, we performed H&E staining (Fig. 3A) and serology (ALT, AST, and cholesterol), which showed that FOS+Pro treatment protected the livers of treated mice from western diet-induced pathological changes. The FOS+ Pro mice showed the lowest NAS scores (3.58 ± 2.1) among all the western diet-fed groups (Fig. 3B). The FOS + Pro mice showed 108%, 68%, 83.6%, 61.8%, 64.9%, and 30.7% lower NAS scores compared to WC, LAC, LAC+ Pro, LAT, LAT+ Pro, and FOS mice, respectively. Furthermore, FOS+ Pro mice showed significantly lower steatosis, hepatitis (inflammation), ballooning, and fibrosis scores, which were 1.14 ± 0.69, 1.42 ± 0.78, 1.16 ± 0.75, and 1.71 ± 1.11 (Fig. 3C). The FOS+ Pro group also showed improved liver enzyme and cholesterol levels in serum, where serum cholesterol levels decreased significantly and ALT levels were found to be the lowest among all the western diet groups (Fig. 3D-F).

### Synbiotic treatment with a combination of fructooligosaccharides and probiotics attenuated western diet-induced inflammatory markers in the livers of mice

**Fig. 4.**
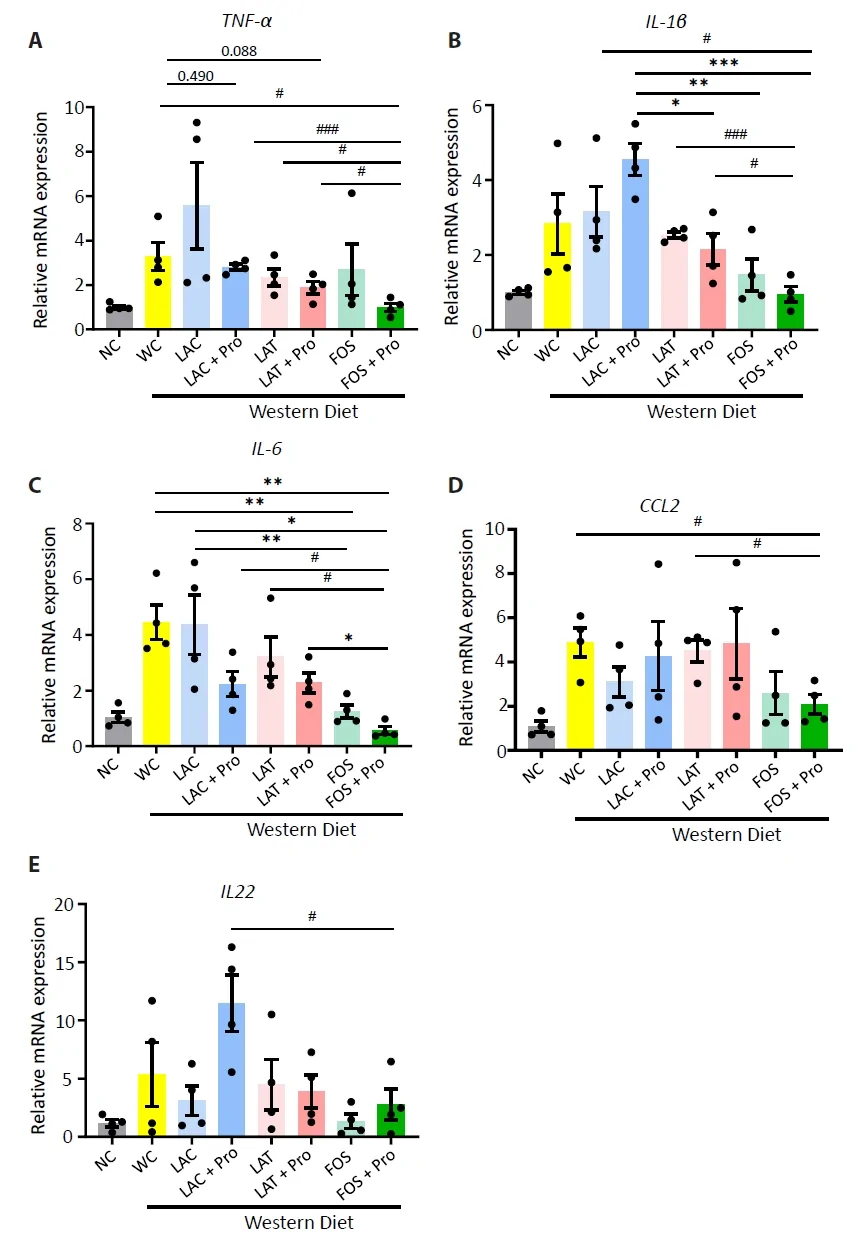
Synbiotic treatment with combination of fructooligosaccharides and probiotic attenuated western diet induced inflammatory markers in liver. Relative mRNA expression of inflammatory genes showed significant downregulation in FOS+Pro group mice. (A) *TNF-α*, (B) *IL-1β*, (C) *IL-6*, (D) *CCL2*, (E) *IL22*. Relative mRNA expression data showed as mean ± SEM and difference between the mean was analyzed by ANOVA with tukey's post hoc test and represented by; *p<0.05, **p<0.001, ***p<0.0001. The mean difference between the two specific groups were analyzed with unpaired parametric student’s t-test and represented by #*p*<0.05, ##*p*<0.01, ###*p*<0.001.

The long-term intake of the western diet induced inflammation in the livers of the mice, which is a critical factor in MASLD progression. TNF-α, a key regulator of inflammatory responses in MASLD, showed lower expression levels in all the synbiotic groups compared to the respective prebiotic-only groups (Fig. 4A). The FOS+ Pro-treated group showed a significant decrease in TNF-α expression compared to the WC, LAC, LAC+ Pro, and LAT groups and a non-significant decrease compared to the LAT+ Pro and FOS groups. Similarly, MASLD progression-related interleukins and chemokines also showed decreased expression in the FOS+ Pro treated mice (Fig. 4B-E). The FOS+ Pro-treated mice showed significantly lower expression levels of IL-1β and IL-6 compared to mice in the WC, LAC, LAC+ Pro, LAT, and LAT+ Pro groups (Fig. 4B, C). The C–C motif chemokine ligand 2 (CCL2) also showed a downregulation trend in the livers of FOS+ Pro-group mice and was significantly decreased compared to the WC and LAT groups and non-significantly decreased compared to the LAC, LAC+ Pro, and FOS groups (Fig. 4D). Unlike IL-1β and IL-6, the IL-22 expression levels did not change much in FOS+ Pro treatment group (Fig. 4E).

### Synbiotic treatment with a combination of fructooligosaccharides and probiotics downregulated metabolic regulator genes in the livers of western-diet-fed mice

**Fig. 5.**
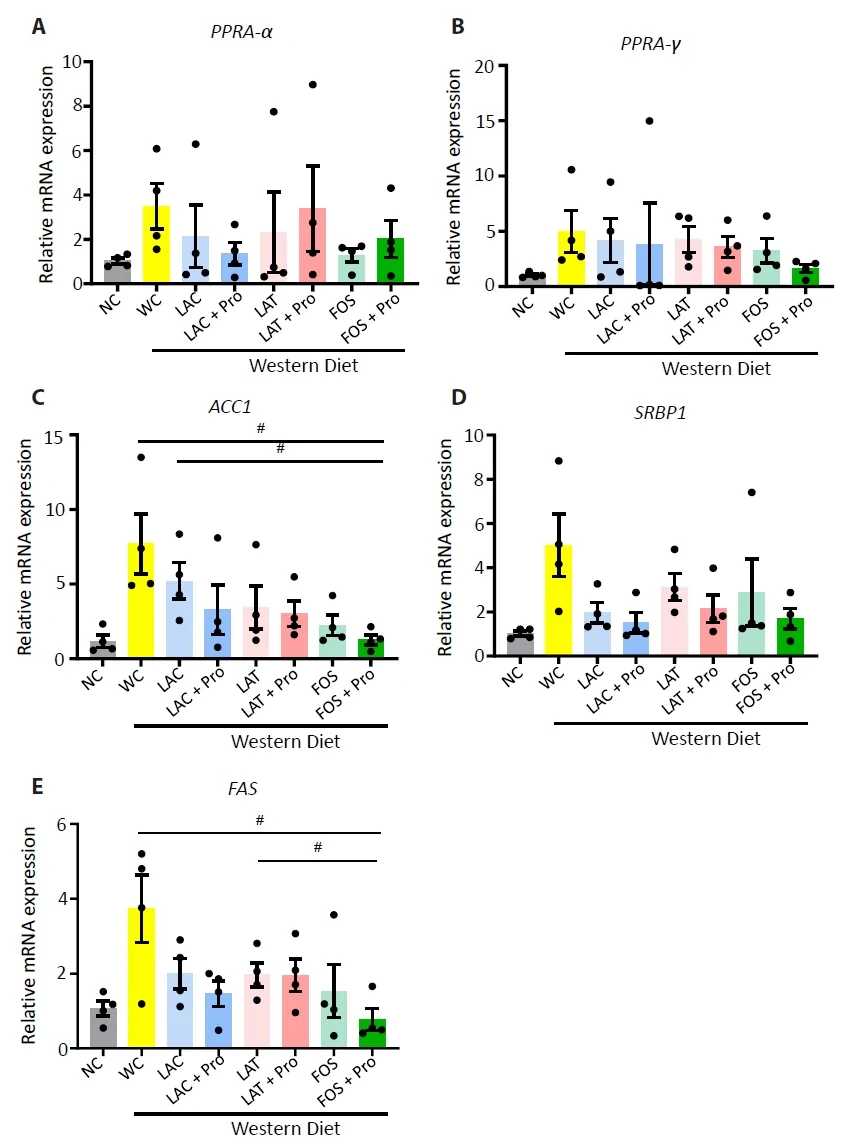
Synbiotic treatment with combination of fructooligosaccharides and probiotic abated metabolic regulator genes in western diet fed mice liver. The metabolic regulator gene exhibited the lowered expression in FOS+Pro group mice liver. (A) *PPRA-α*, (B) *PPRA-γ*, (C) *ACC1*, (D) *SRBP1*, (E) *FAS*. Relative mRNA expression data showed as mean ± SEM and difference between the mean was analyzed by ANOVA with tukey's post hoc test and represented by; *p<0.05, **p<0.001, ***p<0.0001. The mean difference between the two specific groups were analyzed with unpaired parametric student’s t-test and represented by #*p*<0.05, ##*p*<0.01, ###*p*<0.001.

Typically, expression of genes related to lipid metabolism increases in MASLD. Therefore, we measured the expression of lipid metabolism-regulating genes in the treatment group mice (Fig. 5A-E). The FOS + Pro mice exhibited decreased mRNA expression of peroxisome proliferator-activated receptors (alpha and gamma) (Fig. 5A, B), particularly of PPRA-γ, which had the lowest expression levels among all the western diet-fed groups. The acetyl-CoA carboxylase 1 (AAC1) gene also showed the lowest mRNA expression the FOS + Pro mice, among all the western diet-fed groups, and showed significant reduction compared to WC and LAC groups (Fig. 5C). The sterol regulatory element-binding protein 1 (SREBP1) gene was downregulated in all the treatment groups compared with the WC group, but the differences between the WC and treatment groups were not significant (Fig. 5D). Fatty acid synthase (FAS), which is responsible for de novo lipogenesis, was significantly reduced in the FOS + Pro mice compared to the WC and LAC mice (Fig. 5E) and represented the lowest mRNA expression among all the groups including NC.

### Combined treatment with fructooligosaccharides and probiotics downregulated lipid transport-associated genes in the livers of western diet-fed mice

Liver steatosis is the foremost and initial pathophysiology of MASLD, and CD36 is a key gene involved in steatosis. In particular, the CD36 protein is responsible for long-chain fatty acids uptake in liver cells and increases triacylglycerol storage in the liver. The CD36 gene expression in the liver tissues was significantly lower in the FOS + Pro mice than in the WC and LAT mice. A similar downregulation of CD36 expression was observed in the LAC+ Pro mice. Although both of these synbiotic treatment groups (FOS+Pro and LAC+ Pro) presented similarly decreased CD36 expression levels, the FOS+Pro mice had the lowest CD36 expression level among all the western diet-fed mice.

The gene encoding CYP1B1 in the cytochrome P450 family has an essential role in lipid metabolism and is upregulated in the liver when diets contain high levels of fats. The FOS+Pro treatment downregulated the expression of CYP1B1 to the lowest level measured among all the western diet-fed mice. The gene expression of CYP1B1 was significantly downregulated in the FOS+Pro group when compared to the WC, LAT and LAT+ Pro groups and non-significantly downregulated when compared to the LAC, LAC+ Pro and FOS groups.

## Discussion

This study provides compelling comparative evidence that a long-term synbiotic treatment combining fructooligosaccharides (FOS) and probiotics (Pro) had better efficacy than other synbiotic combinations (lactitol and lactulose) for limiting the adverse effects of the western diet used to induce MASLD in mice. Our findings of improved clinical markers have highlighted the potential of a FOS+Pro synbiotic approach in mediating MASLD-associated inflammatory pathways and attenuating disease progression.

The comparative human microbiome analysis of healthy and MASLD patients revealed a significant decline in abundances of *Bifidobacterium* and *Lactobacillus* genera as disease severity progressed. These genera are well-known for their health-promoting gut-microbial species, which produce beneficial metabolites to improve the metabolic profile and overall health of humans (Ghosh et al., 2022; Ross et al., 2024). Similar dysbiosis has been reported in previous studies, where decreases in these beneficial bacteria were related to pathogenesis of NAFLD via disrupted gut barrier function and increased inflammation (Aron-Wisnewsky et al., 2020; Lee et al., 2020). Most well-known probiotic species belong to these two genera, which have been used in multiple studies and have been shown to improve liver functions (Swanson et al., 2020). Based on the results of previous studies, six bacterial species were selected for this study, including four from *Lactobacillus* (*L. acidophilus*, *L. reuteri*, *L. fermentum*, and *L. lactis*) and two from *Bifidobacterium* (*B. breve* and *B. longum*). Similarly, lactitol, lactulose and FOS were selected as prebiotics already known to improve liver disease.

Based on the previous works discussed above, a mouse experiment was designed to evaluate the potential of synbiotic treatment in mitigation of MASLD. Overall, the synbiotic combination FOS+Pro showed the best efficacy in preventing western diet-induced phenotypical changes in mice, including body weight gain and liver enlargement, compared to the other prebiotics and matching synbiotic combinations. The synergistic effect of FOS+Pro indicates that FOS may have enhanced colonization and activity of the administered probiotic, which restricted the weight-gain effects of the western diet (Fig. 2). Western dietary intake distorts liver cellular architecture, which can be observed through the NAFLD activity score (NAS) and other histological parameters (steatosis, inflammation, ballooning, and fibrosis)(Hoogerland et al., 2022; Vacca et al., 2024). In the study reported here, these histological parameters improved more in the FOS+Pro group than in the other treatment groups. Positive impacts of improved cellular integrity were reflected in serum markers such as ALT, AST and cholesterol, where FOS+Pro synbiotic-treated mice showed improvement in serum marker levels, particularly in ALT. Collectively, these findings indicate that combining probiotics with FOS as a synbiotic treatment for MASLD had the highest potential for restoring liver cellular integrity and functionalities. Additionally, combining probiotics with FOS enhanced the protective effect of FOS against western diet-induced MASLD.

Inflammatory pathways are directly linked to MASLD pathogenesis and/or progression of severity (Hoogerland et al., 2022; Peiseler et al., 2022). Proinflammatory cytokines such as TNF-α, IL-1β, and IL-6 have pivotal roles in exacerbating hepatic inflammation, fibrosis, and insulin resistance (Sutti and Albano, 2020). These proinflammatory cytokines promote a self-expanding inflammation cycle, which drives the inflammatory cascade in the hepatic microenvironment. Proinflammatory cytokines such as TNF-α and IL-1β accelerate hepatocytic injury and hepatic stellate cell (HSC) activation. Intensification of HSC activation initiates the alternative hepatic microenvironment repair pathway, which leads to scarring of the liver and escalates to liver fibrosis. Combining probiotics with FOS improved TNF-α and IL-1β expression compared to FOS treatment alone and presented expression levels similar to those of the normal control group. Additionally, IL-6 is conventionally recognized as a proinflammatory cytokine but has a complicated dual nature in MASLD. In early disease stages, IL-6 has shown protective effects but in later stages has contributed to disease progression. In the study presented here, FOS+Pro treatment normalized IL-6 expression in liver tissues more effectively than FOS alone or the treatments applied in the other treatment groups. Interestingly, supplementing all three prebiotics (lactitol, lactulose and FOS) with probiotics improved TNF-α and IL-6 expression in liver tissues more than prebiotics alone, which showed that the synbiotic combinations of prebiotics and probiotics exhibited strengthened proinflammatory cytokine-suppressing effects. Inflammatory responses are further intensified by chemokines such as CCL2 that facilitate inflammatory cell recruitment into the liver. Therefore, decreased proinflammatory cytokine expression led to lower chemokine levels in liver and eased the inflammatory response. The FOS+Pro treatment improved CCl2 expression more than FOS alone, indicating that adding probiotics alongside FOS improved the FOS treatment’s capability for narrowing the western-diet triggered inflammatory cascade.

These observed improvements in expression of inflammatory mediators influenced the metabolic regulatory profile through intricate linkages. Among various metabolic regulators, ACC1 and FAS are decisive enzymes in de novo lipogenesis, primarily contributing to lipid accumulation, and are typically upregulated in MASLD (Cao et al., 2023; Xu et al., 2022). Their interdependent linkage with the proinflammatory cytokines TNF-α and IL-1β is well established. Increased TNF-α and IL-1β enhance lipogenesis via upregulating expression of the transcription factor SREBP-1c, which predominantly regulates ACC1 and FAS expression. In contrast, the inhibition of ACC1 and FAS minimizes hepatic inflammation and decreases expression of inflammatory markers such as IL-6 and CCL2. Additionally, increased IL-22 expression modulates lipid metabolism genes, including ACC1 and FAS, which shows hepatoprotective effects by reducing oxidative stress. These complex cross-linkages among lipogenic enzymes (ACC1 and FAS) and inflammatory modulators emphasize the multifaceted nature of MASLD progression (Horn and Tacke, 2024). In the present study, adding probiotics to the FOS treatment improved the expression of both lipogenic enzymes (ACC1 and FAS) in the liver, possibly reducing lipogenesis and leading to reduced steatosis and liver inflammation. Furthermore, the FOS+Pro combination substantially reduced the expression of CD36 and Cy1b1, which are both crucially involved in MASLD progression and aggravation. CD36 palmitoylation at the plasma membrane intensifies fatty acid translocation and accumulation in hepatocytes, exacerbating steatosis. Moreover, CD36 activation triggers the JNK inflammatory cascade, which increases the production of proinflammatory cytokines, including TNF-α and IL-1β (Rada et al., 2020). This study revealed that combining probiotics with FOS improved CD36 expression in the liver and restrained MASLD aggravation. Similarly, FOS+Pro also normalized Cyp1b1 expression, which has been linked to oxidative stress, lipid peroxidation and inflammatory modulation. Cyp1b1 and IL-6 are interconnected and contribute to a feed-forward loop of inflammation and oxidative stress that worsens MASLD.

This comparative analysis of three synbiotic treatments and their corresponding prebiotics revealed a few key results, such as the superior capabilities of the FOS+ Pro combination in preventing western diet-induced MASLD. Among all three prebiotics (lactitol, lactulose and FOS), FOS exhibited excellent MASLD inhibition ability, and synbiotic treatment with FOS+Pro further improved efficacy in restraining MASLD. Empirically, prebiotics are expected to improve gut microbial diversity and increase the abundance of health-promoting microbiomes, and FOS significantly improves gut microbial diversity. Moreover, studies have shown that FOS enhances fecal SCFAs and improves lipid metabolic and inflammatory markers, effects that are analogous to the results of this study. This study presents new elaborate and substantial evidence that synbiotic treatment with FOS+Pro increased the effectiveness of FOS in treating MASLD. This study highlights the molecular mechanism through which synbiotic FOS+Pro lowered hepatic inflammation more effectively than FOS alone, other prebiotics alone, and other synbiotic combinations. Inflammatory alteration led to the modulation of key lipid metabolism pathways and restrained the progression of MASLD, as has been observed in various human clinical trials. However, this study has various limitations, including a lack of evidence to explain why the other two synbiotic combinations failed to produce MASLD-protective results. Furthermore, this study did not compare the gut microbial changes produced by FOS+Pro treatment to changes previously observed with FOS treatment, which has been shown to improve the gut microbial ecology, increase fecal SCFAs and limit the progression of MASLD.

## Figures and Tables

**Fig. 6.**
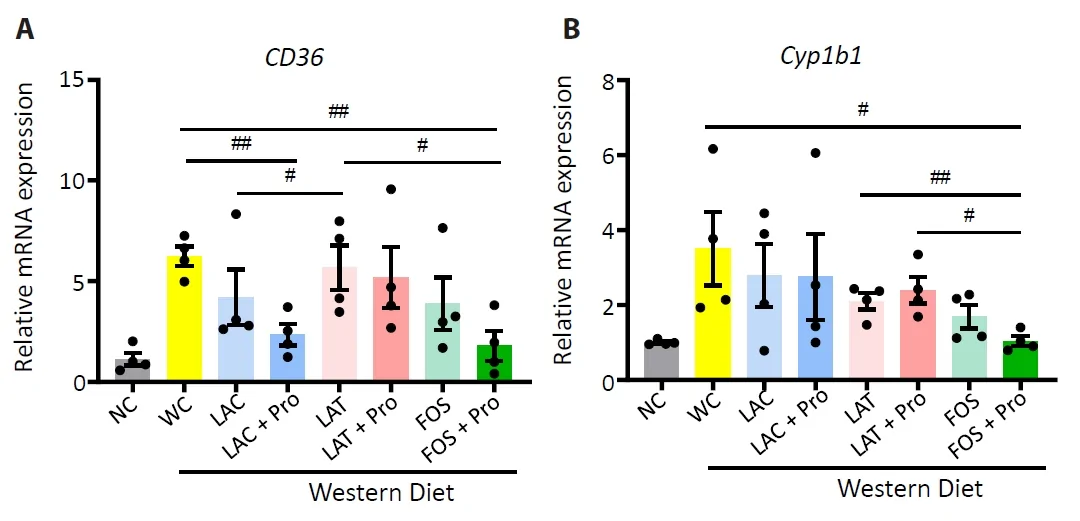
Combined treatment with fructooligosaccharides and probiotic abated lipid transport associated genes in western diet fed mice liver. The gene associated with fatty acid transportation showed lowering in the mRNA expression in FOS+Pro group mice liver. (A) *CD36*, (B) *Cyp1b1*. Relative mRNA expression data showed as mean ± SEM and difference between the mean was analyzed by ANOVA with tukey's post hoc test and represented by; *p<0.05, **p<0.001, ***p<0.0001. The mean difference between the two specific groups were analyzed with unpaired parametric student’s t-test and represented by #*p*<0.05, ##*p*<0.01, ###*p*<0.001.

**Table 1.** Patients’ baseline characteristics

Parameters	HC	MAFLD	MASH
Mean (SD)	(n=18)	(n=18)	(n=14)
Age (years)	59.7 (10.1)	57.0 (11.5)	57.5 (17.3)
Female (n, %)	10 (56%)	14 (78%)	4 (29%)
BMI (kg/m^2^)	21.7 (1.7)	27.6 (1.1)***	28.2 (1.5)***
AST (IU/L)	23.4 (5.3)	58.3 (28.9)***	52.7 (8.2)***
ALT(IU/L)	20.8 (9.9)	61.2 (48.3)***	64.6 (12.1)***
Cr (mg/dL)	0.87 (0.17)	0.82 (0.2)	0.98 (0.11)^#^
Cho (mg/dL)	196.4 (44.8)	216.1 (57.2)	230.0 (57.0)
HDL (mg/dL)	60.5 (19.7)	53.0 (9.5)	47.6 (14.7)
TG (mg/dL)	134.9 (157.8)	184.1 (92.0)	168.0 (24.6)
γ-GT (IU/L)	36.7 (49.5)	76.3 (46.5)^#^	204.8 (348.1)*

HC, healthy control; MAFLD, Metabolic dysfunction-associated fatty liver disease; MASH, Metabolic dysfunction-associated steatohepatitis; n, number; BMI, body mass index; AST, aspartate aminotransferase; ALT, alanine aminotransferase; Cr, creatinine; Chol, cholesterol; γ-GT, gamma glutamyl transpeptidase. The statistical difference between groups mean value was measured by ANOVA; *p<0.05, and individual difference measured by two groups evaluated by t-test using Mann-Whitney test compare rank test ^#^*p*<0.05.

**Table 2.** List of primers used in this study

Gene name	Forward primer (5’ to 3’)	Reverse primer (5’ to 3’)
*PPIA*	GAGCTGTTTGCAGACAAAGTTC	CCCTGGCACATGAATCCTGG
Tnf-α	CCCCAAAGGGATGAGAAGTT	CACTTGGTGGTTTGCTACGA
Il-1β	GAAATGCCACCTTTTGACAGTG	TGGATGCTCTCATCAGGACAG
Il-6	TAGTCCTTCCTACCCCAATTTCC	TTGGTCCTTAGCCACTCCTTC
Ccl2	TCCCAATGAGTAGGCTGGAG	TCTGGACCCATTCCTTCTTG
Ppar-α	AGAAGTTGCAGGAGGGGATT	TCGGACTCGGTCTTCTTGAT
Ppar-γ	GGTGTGATCTTAACTGCCGGA	GCCCAAACCTGATGGCATTG
*SEBP1*	TACTTCTTGTGGCCCGTACC	TCAGGTCATGTTGGAAACCA
*FAS*	AAGCGGTCTGGAAAGCTGAA	AGGCTGGGTTGATACCTCCA
*ACC1*	CAGTAACCTGGTGAAGCTGGA	GCCAGACATGCTGGATCTCAT
*CD36*	TGGCCAAGCTATTGCGACAT	ACACAGCGTAGATAGACCTGC
*Cyp1b1*	GAATCATGACCCAGCCAAGT	TAATGAAGCCGTCCTTGT CC
*IL22*	TTTCCTGACCAAACTCAGCA	TCTGGATGTTCTGGTCGTCA
